# Low-Cost Pathology Signals for Risk Stratification in High-Risk Non-Muscle-Invasive Bladder Cancer: A Narrative Review

**DOI:** 10.3390/cancers18142269

**Published:** 2026-07-15

**Authors:** Núria Sala-González, Sviatoslav Chekhun, Claudia Fina, Marina Vilaseca, Olha Rossylna, Roger Boix, Berta Bella-Burgos, Josep Comet

**Affiliations:** 1 Catalan Institute of Oncology, Avenida Francia, s/n, 17007 Girona, Spain; 2 Girona Biomedical Research Institute, 17007 Girona, Spain; 3 Institute of Economic and Legal Research, National Academy of Sciences of Ukraine, 01001 Kyiv, Ukraine; 4 Josep Trueta University Hospital, 17007 Girona, Spain

**Keywords:** non-muscle-invasive bladder cancer, T1 high-grade, tumour budding, T1 substaging, lamina propria invasion, E-cadherin, CDH1, BCG failure, risk stratification, radical cystectomy

## Abstract

T1 high-grade (T1HG) urothelial carcinoma of the bladder presents a persistent clinical challenge: despite uniform high-risk classification under EAU guidelines, BCG failure and disease progression rates range from 10% to 40% across published series. Standard clinicopathological variables do not adequately explain this heterogeneity. Three pathological parameters evaluable from routine TURBT specimens—T1 substaging by lamina propria invasion depth, tumour budding at the invasion front, and E-cadherin (CDH1) immunohistochemistry—share a common mechanistic basis in CDH1-driven partial epithelial-to-mesenchymal transition and may refine escalation-oriented risk stratification without requiring additional tissue or molecular testing. We conducted a narrative critical review of PubMed/MEDLINE (January 2000–February 2026; 28 included studies) to evaluate the quantitative evidence for each parameter, with emphasis on reproducibility and BCG-specific outcome data. T1 substaging carries the strongest evidence: pooled progression HR 3.29 (95% CI 2.39–4.51) across 36 studies (n = 6781), with BCG failure of 41% vs. 21% in a centralised BCG-treated registry cohort of 264 patients on multivariable analysis. Tumour budding shows consistent adverse associations in BCG-treated pT1 NMIBC; zero progression was observed in the low-budding subgroup in the only available BCG-specific full-text cohort. CDH1 IHC is directionally supportive but limited by scoring heterogeneity (I^2^ = 63%). All three parameters are mechanistically coherent and assessable from routine TURBT slides. Prospective validation with pre-specified thresholds and standardised scoring protocols is required before clinical implementation can be recommended.

## Simple Summary

High-grade bladder tumours that have not yet invaded the muscle wall are difficult to manage: some are cured by BCG immunotherapy, while others progress despite treatment and may require removal of the bladder. At present, doctors cannot reliably predict which tumours will respond. This review examines three features that pathologists can already assess on the routine tissue sample taken during bladder tumour resection, how deeply the tumour has grown into the tissue beneath the surface lining, whether tumour cells break away as tiny clusters at the invading edge, and the loss of a protein that normally holds cells together. We show that these three features are biologically connected and that the depth of invasion, in particular, is linked to a higher risk of BCG failure. None of these markers is yet ready to change treatment on its own, but reporting them could help medical teams identify higher-risk patients earlier. We outline what future studies would need to confirm their value, so that these simple, low-cost measurements might eventually guide more personalised treatment decisions.

## 1. Introduction

T1HG UCB occupies an uncomfortable position clinically. Although the EAU guidelines classify it uniformly as high-risk, the actual clinical trajectory varies considerably between patients. Muscle-invasive progression occurs in 10–40% of cases across published series, and any high-grade recurrence after a full course of BCG is observed in substantially more patients. Both figures depend on cohort composition, follow-up duration, and endpoint definitions, but the spread in either metric reflects genuine biological heterogeneity rather than methodological noise alone [1,2,3]. The central treatment dilemma follows directly from this heterogeneity. When a patient progresses after a delayed cystectomy, survival outcomes are worse than with earlier surgery [4,5]. Yet RC carries irreversible morbidity, and a meaningful proportion of T1HG patients who undergo upfront surgery would probably have responded to intravesical therapy. Once BCG has failed to control disease, the therapeutic landscape becomes substantially more complex [6,7].

Part of what drives aggressive behaviour in T1HG UCB is the partial dismantling of cell-to-cell adhesion at the invasion front. Transcriptional suppression of CDH1 through ZEB1/ZEB2 or SNAI1 weakens E-cadherin-dependent junctions without necessarily producing fully mesenchymal cells [8]. The resulting phenotype remains partially epithelial, permitting collective stromal infiltration. On haematoxylin and eosin (H&E) sections, this may be read as tumour budding, and it tends to accompany deeper LP penetration [9,10,11]. CDH1 is one of several molecular axes involved in this process; the focus here reflects the available literature rather than a claim that it is uniquely explanatory.

Substantial observational evidence links LP invasion depth and tumour budding to adverse outcomes in T1HG NMIBC. Yet neither parameter is reported consistently in routine TURBT pathology, and no prospective study has been powered to test whether substaging or budding status predicts BCG failure in BCG-treated T1HG cohorts. Existing reviews have generally examined these parameters in isolation or focused on composite statistical risk models (EORTC, CUETO, EAU) rather than on the individual pathological features visible on the TURBT specimen. This review makes two distinct contributions. First, it integrates T1 substaging, tumour budding, and CDH1 immunohistochemistry within a single partial-EMT biological framework, showing why these three morphological parameters are mechanistically linked rather than independent. Second, and as the principal practical output, it translates that integrated evidence into an explicit, reproducibility-aware framework for multidisciplinary team (MDT) discussion, calibrated to what the current evidence genuinely supports and explicit about the prospective validation still required. Throughout, we distinguish carefully between prognostic association and predictive utility, and between what may reasonably inform discussion now and what would be needed before any parameter could formally alter management.

## 2. Materials and Methods

### 2.1. Search Strategy and Review Framework

This study was conducted as a structured narrative review with a systematic search strategy, following the SANRA framework for narrative review quality assessment [12]. The review was not registered in PROSPERO. A PRISMA-adapted study selection flow and SANRA quality assessment are provided as Supplementary File S3.

**Figure 1:**
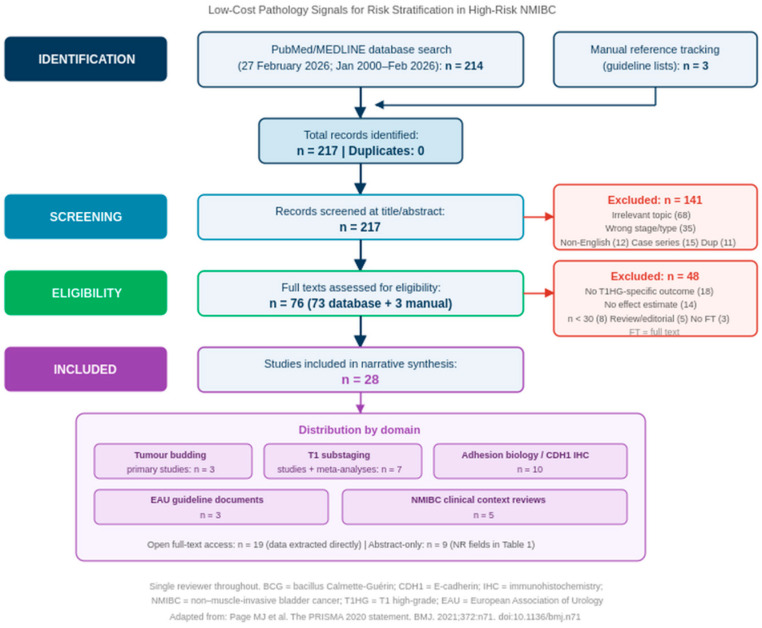
Literature search and study selection flow diagram (PRISMA 2020 [13]).

PubMed/MEDLINE was searched on 27 February 2026 for publications from 1 January 2000 onwards. Search terms combined Medical Subject Headings (MeSH) and free-text terms for NMIBC, T1 urothelial carcinoma, tumour budding, T1 substaging, lamina propria invasion, muscularis mucosae, E-cadherin/CDH1 and related cadherin isoforms, and oncological endpoints including recurrence, progression, BCG failure, and cancer-specific survival (CSS). The full search string is provided in Supplementary File S1. The search returned 214 candidate records, and Figure 1 summarises the selection process.

### 2.2. Eligibility Criteria and Data Extraction

We included peer-reviewed original studies and evidence syntheses reporting at least one quantitative oncological endpoint—recurrence-free survival (RFS), high-grade RFS (HG-RFS), progression-free survival (PFS), cancer-specific survival (CSS), or BCG failure rate—with effect estimates, confidence intervals, and a study population of at least 30 patients. Editorials and case reports were excluded. Screening and data extraction were performed by a single reviewer (S.C.), consistent with the narrative design of this review; extracted quantitative values were subsequently cross-checked against the original publications by a second author (N.S.), and any discrepancies were resolved by consensus with reference to the primary source. Where open-access full text was available, data were extracted directly. Abstract-only records were included only when the reported endpoint, effect estimate, and sample size were unambiguously stated in the abstract and no open full text was accessible; such records were retained to avoid selective exclusion of otherwise eligible data, and all variables not explicitly reported were annotated as “not reported” (NR) in Table 1 so that the completeness of each source is transparent to the reader.

A narrative rather than pooled synthesis was used because IHC scoring protocols, budding threshold definitions, and clinical endpoints differed substantially across the retrieved studies; I2 values are reported throughout to make the extent of that heterogeneity explicit rather than buried.

### 2.3. Clinical Questions and Quality Appraisal

The review was organised around three clinical questions: (1) whether T1 substaging by LP invasion depth provides independently prognostic risk discrimination in BCG-treated T1HG UCB and predicts BCG failure; (2) whether tumour budding at the invasion front is a reproducible independent prognostic parameter for progression, CSS, and upstaging; and (3) whether reduced CDH1 expression by IHC offers clinically actionable information or whether current scoring heterogeneity limits it to biological contextualisation.

Retrospective cohort quality was appraised using the QUIPS tool [20] and the two included meta-analyses were assessed against AMSTAR-2 criteria [21]. A quality appraisal table is provided in Supplementary File S2.

## 3. Results

### 3.1. Overview of the Evidence Base

Twenty-eight studies met inclusion criteria. Table 1 summarises the index studies with primary quantitative outcome data across the three domains. The remaining citations are integrated narratively below.

#### 3.1.1. Tumour Budding

Three published studies have evaluated tumour budding in T1 UCB, all retrospective single-centre series [9,10,14]. This limited evidence base should be acknowledged at the outset before engaging with the data.

Fukumoto et al. examined 121 T1 bladder cancers and found that budding was an independent predictor of stage progression on multivariable analysis, with a 5-year progression-free rate of 91% in budding-negative versus 62% in budding-positive tumours [9]. The magnitude of the difference is clinically meaningful, and the multivariable analysis is reassuring in a setting in which confounding is inevitable. However, the bud-count threshold was derived and tested within the same cohort.

Eckstein et al. used a hotspot-based approach at ×20 magnification in 92 pT1 NMIBC specimens, defining a bud as any single cell or cluster of up to five cells at the invasion margin [10]. Higher bud counts predicted worse RFS, PFS, and CSS. Within the BCG-treated subgroup (*n* = 65), patients with bud counts below the cohort median recorded no progression events and no cancer-specific deaths during follow-up. This is noteworthy, not because it is definitive, but because it is the first suggestion in this literature that low budding might identify a genuinely low-risk BCG-treated subgroup rather than simply a less adverse one. Sixty-five patients from a single institution are not a sufficient evidence base on which to act, but they are sufficient grounds for designing a prospective validation study.

Busquets and colleagues reported an association between budding and the need for early RC in a Spanish series of 95 pT1HG cases, but the analysis was available in abstract form only, precluding extraction of effect sizes [14]. Taken together, the budding literature in UCB remains small, geographically limited, and methodologically heterogeneous, and it has never been subjected to formal inter-observer reliability assessment. The signal is consistent; the infrastructure required for clinical implementation is not yet in place.

#### 3.1.2. T1 Substaging

The strongest quantitative evidence for substaging comes from the meta-analysis by Parizi et al., which pooled 6781 patients from 13 studies across several substaging systems [11]. The headline figure—a pooled HR for progression of 3.29 (95% CI 2.39–4.51)—comes specifically from the depth/metric substaging subgroup, which was the most methodologically homogeneous component of the analysis. Overall heterogeneity for progression was moderate (I^2^ = 47%) under a random-effects model, prediction intervals were not reported, and the full meta-analysis includes both muscularis mucosae (MM)-based and metric systems that capture related but not identical pathological features. The pooled HR of 3.29 is the most defensible summary estimate available but it should not be interpreted as a calibrated threshold transferable across centres using different substaging definitions.

The clinically most relevant question is whether substaging predicts BCG failure in patients who actually received BCG. This is addressed most directly by de Jong et al. [15]. In that registry cohort of 264 high-risk NMIBC patients who completed at least five BCG induction instillations, specimens underwent centralised pathological re-review and were categorised into microinvasive and extensive LP invasion groups. BCG failure occurred in 41% of the extensive group versus 21% of the microinvasive group (*p* = 0.002), and the three-year HG-RFS rate was 64% versus 83% (*p* = 0.004). On multivariable analysis, adjusted for concomitant carcinoma in situ (CIS), multifocality, and prior BCG, substaging remained independently associated with both HG-RFS (HR 3.2, *p* = 0.005) and PFS (HR 3.0, *p* = 0.009). Particularly informative was the discrimination observed within the highest EAU risk group: patients meeting those criteria but showing only microinvasive disease had markedly better PFS than those plus extensive invasion (*p* = 0.038).

One practical issue cuts across all MM-based substaging: the muscularis mucosae is identifiable in only approximately 69% of TURBT specimens [16]. Metric substaging, classifying invasion as microinvasive when the tumour front lies within 0.5 mm of the urothelial basement membrane and extensive when it extends beyond that distance, is independent of MM visibility and has been shown feasible in a multicentre setting of 601 patients by Fransen van de Putte and colleagues [16]. Orsola et al. demonstrated in a 20-year prospective series of 132 patients that LP depth substaging discriminated outcomes regardless of MM status [18]. Data from Soukup et al. [17] and Holmäng et al. [19] extend the prognostic gradient associated with invasion depth across three decades and several substaging systems. The 2022 EAU guideline recommendation to report substaging is a Weak recommendation (LE 3). This reflects expert consensus rather than prospective validation, but the field’s position is clear: this information is clinically relevant [2].

A dimension largely absent from the published substaging literature is the relationship between substaging at initial TURBT and substaging at re-TURBT. EAU guidelines recommend re-TURBT in T1HG as standard practice, and pathological findings at re-TURBT—including residual T1 disease, upstaging to T2, and substaging at re-TURBT—carry independent prognostic information [1,2]. None of the substaging studies reviewed here systematically addresses whether initial and re-TURBT substages correlate, or which is more predictive of BCG failure. De Jong et al. do not specify whether substaging was performed on initial or re-TURBT specimens [15]. This is not a peripheral methodological detail; it affects how any prospective validation study should be designed.

#### 3.1.3. Adhesion Biology and CDH1 Expression

The observation that CDH1 loss, tumour budding, and deeper LP invasion tend to co-occur in the same specimens is biologically coherent rather than coincidental. In urothelial cancer, transcriptional repressors such as ZEB1/ZEB2 and SNAI1 can suppress CDH1, dismantling E-cadherin-dependent adherens junctions [8]. The resulting state is more accurately described as partial epithelial–mesenchymal transition (EMT) than complete mesenchymal conversion. Cells retain enough epithelial identity to maintain cohesion in small clusters, but they become sufficiently weakly adherent to migrate collectively through stroma. That collective migration is what pathologists recognise morphologically as budding, and the capacity for stromal infiltration is what produces a broader and deeper invasion front. Bryan’s review provides a useful framework for these relationships in urothelial cancer [8], although experimental data in T1-specific models remain limited, and the relative contribution of CDH1 suppression versus other adhesion-disrupting events is not fully resolved. CDH1 loss should therefore be viewed as the best-characterised mechanism in this context rather than the sole molecular explanation.

Cadherin switching—CDH1 loss paired with CDH2 upregulation—has been associated with shorter intravesical RFS after TURBT in a mixed-stage NMIBC cohort of 116 patients (log-rank *p* = 0.02) [22]. N-cadherin [23,24] and P-cadherin [25] also show directionally consistent prognostic signals in urothelial tumours, and catenin expression data point the same direction [26,27]. A tissue microarray study combining CDH1 IHC with T1a/b substaging found additive prognostic discrimination, consistent with the underlying biology, although confirmation in larger series with explicit BCG-treatment data is needed [28]. Taken together, these studies show that the adhesion disruption visible on H&E as budding has a molecular correlate measurable by IHC. The unresolved question is whether measuring that correlate provides information beyond what the morphology already conveys.

Xie et al. pooled 18 studies of CDH1 expression in UCB and found associations with both recurrence (odds ratio [OR] 2.91, 95% CI 1.89–4.47) and progression (OR 3.44, 95% CI 2.09–5.65) [29]. The central problem is heterogeneity (I^2^ of 63%) which reflects a literature built on three different antibody clones (NCH-38, 36B5, SP1), H-score cut-offs ranging from 50 to 200, mixed tumour stages, and no inter-laboratory reference standards. A pooled OR generated under those conditions is directionally informative, but it does not yield a scoring threshold that a pathologist in a different laboratory, using a different antibody clone, could apply reproducibly to the same patient. Shariat et al. showed that reduced E-cadherin predicts CSS in CIS [30], and Lipponen and Eskelinen demonstrated an association with invasive disease and recurrence in a 1995 cohort [31]. Both findings are consistent with the overall direction, but neither study used contemporary substaging or BCG-treatment data. For now, CDH1 IHC is better read alongside the morphology than used independently. That mechanistic role is still useful. That mechanistic role is still useful: understanding that budding and deeper invasion are downstream consequences of E-cadherin disruption helps define which tissue compartment should be scored, which antibody clones are biologically relevant, and why the tumour-stroma interface, rather than the tumour core, is the correct region to assess.

## 4. Discussion

### 4.1. Clinical Meaning of the Available Evidence

The evidence reviewed here has direct implications for what is documented in a TURBT pathology report and how T1HG cases are discussed in a multidisciplinary team (MDT) setting. It does not yet change the management algorithm itself, and conflating those two levels of interpretation would be a mistake.

Substaging and budding are prognostic parameters. They indicate that patients with extensive LP invasion or high-grade budding experience worse outcomes on average. That is genuinely useful information for MDT discussions, even in the absence of predictive validation, that is, without evidence that a given parameter identifies differential treatment benefit. No randomised study has tested whether early RC in the extensive-invasion subgroup improves survival compared with BCG induction followed by close surveillance. The 2024 EAU guidelines introduced a very-high-risk T1HG subgroup defined by concomitant CIS, multiple high-grade recurrences within 12 months, prostatic urethral involvement, and variant histology [1,3]. Sylvester et al. identified substaging as one of seven independent predictors within the EAU prognostic factor risk groups [3]. Neither source mandates RC on the basis of substaging alone, and that caution is appropriate given the current evidence. What the available data do support is making the substaging findings explicit at MDT discussion and documenting that they informed the conversation. For many centres, this would represent a practice change that requires no additional financial outlay.

### 4.2. Methodological Constraints

The weaknesses of the evidence base are real and need to be stated clearly. The Parizi meta-analysis is the most frequently cited quantitative anchor for substaging, but I^2^ = 47% under a random-effects model, without prediction intervals, means the summary HR of 3.29 reflects studies measuring related but heterogeneous constructs [11]. It is a directional signal, not a transferable decision threshold.

The tumour budding literature is less mature. Across three retrospective single-centre cohorts, the combined analysed population remains below 350 patients, there are no prospective data, and no inter-observer reproducibility data have been published. A prospective budding study trial in T1HG NMIBC would likely require approximately 150 progression events, implying a BCG-treated cohort of roughly 300–400 patients followed for at least 5 years. That study has not yet been done. Describing budding as validated for clinical use would be inaccurate. What can be said is that the signal is consistent across independent cohorts, biologically plausible, and strong enough to justify prospective validation.

### 4.3. Reproducibility as the Main Barrier to Implementation

Reproducibility is the major bottleneck. This matters more here than in many biomarker discussions because both substaging and budding depend on pathologist judgement applied to morphological features. A parameter that different observers score differently on the same slide cannot credibly inform a decision as consequential as RC.

For MM-based T1 substaging, published kappa values range from 0.48 to 0.72 in dedicated reproducibility studies [32,33]. Metric substaging improves agreement to approximately 0.65–0.81 among trained uropathologists in multicentre settings [16]. That is an advance, but the lower end of that range remains below the level of agreement that would be desirable (≥0.80) before a pathological feature contributes materially to escalation decisions. For tumour budding in UCB specifically, no inter-observer reliability data are available. Fukumoto, Eckstein, and Busquets used different field areas, magnifications, and counting rules, and none reported kappa or intraclass correlation coefficients. This is not a minor omission; it is the single most important methodological obstacle to clinical integration. Solving it will require a formal consensus process with pre-specified reproducibility targets, not merely another retrospective cohort.

Computational pathology is worth evaluating as a route to standardisation. In colorectal and gastric cancer, artificial intelligence-assisted hotspot detection and automated bud quantification have shown high concordance with expert assessment [34]. Systematic evaluation of similar approaches in T1HG UCB is warranted.

### 4.4. Biology Is Coherent; Clinical Utility Must Still Be Proven

CDH1 IHC and tumour budding morphology capture different aspects of the same underlying biological process—partial EMT—which explains why they tend to co-occur and why their combination with substaging is mechanistically coherent rather than arbitrary [8]. Whether a composite model incorporating all three markers improves risk discrimination beyond substaging alone in BCG-treated T1HG disease is a reasonable hypothesis, but current data are insufficient to answer it. The Xie meta-analysis (I^2^ = 63%) demonstrates directional prognostic value for CDH1 loss, but not a usable decision threshold [29]. Until a validated T1HG-specific CDH1 protocol exists, including an agreed antibody clone, cut-off, and internal reference standard, IHC remains a biological adjunct rather than an operational clinical test.

### 4.5. Reporting Now; Designing the Missing Study

Among the three parameters, LP invasion depth has the strongest and most reproducible evidence base and is the most reasonable candidate for inclusion in T1HG TURBT pathology reporting. Where the muscularis mucosae is not identifiable—which is the majority of cases—metric substaging using a 0.5 mm threshold from the urothelial basement membrane is the most practical approach, because it is independent of muscularis mucosae visibility and has been shown to be feasible in multicentre settings [2,16]. Extensive invasion, defined as tumour extending beyond 0.5 mm, can be documented explicitly and flagged for MDT review. We emphasise, however, that even for substaging the supporting evidence is derived from retrospective cohorts, and that adoption into routine reporting should be regarded as a reasonable, evidence-informed option rather than a validated standard of care pending prospective confirmation.

For tumour budding, the evidence base is substantially more limited—three retrospective single-centre series with fewer than 350 patients combined, no prospective validation, and no bladder-specific inter-observer reliability data. We therefore do not recommend routine clinical reporting of tumour budding at this stage. Where budding is assessed within a research setting, the hotspot method at ×20 magnification—counting buds in the single most active field, as described by Eckstein et al. [10]—offers the most reproducible starting point, and a binary split at the cohort median is a workable provisional approach for feasibility studies. The median value reported by Eckstein (0.9 buds per × 20 field) is a starting point for prospective calibration, not a validated threshold, and should not be applied to individual clinical decisions in its current form.

CDH1 IHC is not ready for independent clinical application. No validated scoring threshold exists for T1HG UCB, and the pooled literature combines H-score cut-offs from 50 to 200 across three different antibody clones without consensus [29]. Proposing a new provisional threshold on this basis would be inconsistent with the evidence. Where CDH1 IHC is performed as part of a local research protocol, results should be interpreted against the laboratory’s own validated antibody and staining conditions, ideally with adjacent normal urothelium serving as the internal control. Where substaging and budding are borderline, CDH1 loss may contribute to the biological context; it should remain one data point among several rather than a management trigger [29,30,31]. These distinctions are summarised in Table 2, which outlines how each parameter may inform MDT discussion according to its current level of evidence.

This table addresses the two parameters for which observational evidence is currently available to inform MDT discussion: LP invasion depth and tumour budding. CDH1 IHC is not included as a decision criterion because no validated scoring threshold exists for T1HG UCB. Where performed, findings should be recorded in the pathology report and interpreted as biological context rather than a management trigger. No risk thresholds in this framework have been prospectively calibrated. All management decisions should conform to current EAU guideline recommendations and individual clinical context. 

## 5. Limitations

The most important limitation is the absence of prospective randomised data. No trial has tested whether management decisions informed by LP invasion depth or tumour budding improve survival outcomes in T1HG NMIBC. Both parameters remain prognostic in the observational sense; their predictive validity—whether treatment stratification based on them improves outcomes—has not been established. This distinction should accompany any clinical use of these findings.

The second major limitation is the quality and volume of the primary tumour budding literature. Three retrospective single-centre cohorts with a combined analysed population below 350 patients, no inter-observer reproducibility data, and heterogeneous scoring definitions constitute a thin evidentiary basis for proposing routine clinical reporting. The substaging literature is larger and more mature, but the Parizi meta-analysis still shows moderate heterogeneity (I^2^ = 47%) under a random-effects model and lacks prediction intervals, limiting the transferability of its summary estimates [11].

This review was conducted as a narrative synthesis and was not prospectively registered. The search was limited to PubMed/MEDLINE. Embase was not searched, which may have resulted in the exclusion of relevant records not indexed in MEDLINE. Nine included studies contributed abstract-level data only, introducing potential reporting bias, particularly in favour of positive findings. The methodology indicates that QUIPS and AMSTAR-2 were applied; the detailed quality appraisal is provided in Supplementary File S2.

Molecular subtyping was not covered in this review, and this is a genuine limitation. Transcriptomic subtypes associated with differential BCG response have been described in NMIBC-specific datasets, including the early-stage UCB transcriptomic analysis by Hedegaard et al. [35]. It should also be noted that the molecular taxonomy developed by Sjödahl and colleagues [36] was derived primarily from muscle-invasive UCB; its direct application to T1HG NMIBC requires caution and should not be assumed to transfer without NMIBC-specific validation. A risk stratification framework that incorporates only morphological parameters without acknowledging molecular subtype context is incomplete. Future prospective studies should include transcriptomic profiling alongside substaging and budding to determine whether morphological and molecular parameters are additive or overlapping.

Beyond molecular subtyping, patient-derived organoid (PDO) models are emerging as a complementary experimental platform for biomarker validation in bladder cancer. PDO biobanks have been shown to recapitulate the histopathological and genomic diversity of parental tumours and to retain intratumoural heterogeneity through serial passage, with drug-response profiles that correlate with mutational status and, in some series, with patient clinical outcomes [37,38]. For the parameters considered in this review, PDOs offer a potential route to address questions that retrospective morphological studies cannot: whether the pEMT programme underlying tumour budding and CDH1 loss is functionally linked to BCG resistance, and whether the morphological phenotype observed on the TURBT specimen is preserved and mechanistically tractable in a living model. Integrating morphological risk stratification with PDO-based functional validation and transcriptomic profiling represents a realistic direction for the prospective work outlined below, and may help move these parameters from prognostic association toward mechanistically grounded, personalised treatment stratification.

## 6. Conclusions

T1 substaging and tumour budding are currently the two low-cost pathological parameters best supported for inclusion in routine TURBT reporting in T1HG NMIBC. The meta-analysis by Parizi et al. establishes a consistent prognostic gradient for depth/metric substaging across multiple independent datasets [11]. The cohort reported by de Jong et al. translates that gradient into a BCG failure differential that persists after multivariable adjustment in a realistically sized, BCG-treated population with nearly 6 years of median follow-up [15]. Tumour budding shows marked outcome separation in the only full-text BCG-treated dataset, with zero progression events in the low-budding subgroup. The finding has not yet been replicated, but it is biologically coherent and sufficiently compelling to justify serious prospective evaluation [10].

CDH1 IHC contextualises these morphological findings mechanistically. Its independent clinical utility is currently blocked by scoring heterogeneity that has not been resolved through standardisation [29]. This is a tractable methodological problem rather than an intrinsic limitation of the marker itself. It requires the same kind of deliberate consensus process that enabled the International Tumour Budding Consensus Conference (ITBCC) to move colorectal cancer from scattered single-centre definitions to a workable international standard within a few years of dedicated effort [34].

One practical implication of the framework proposed here concerns patient selection for future clinical trials. Patients with extensive LP invasion and high-grade budding who are not surgical candidates represent a biologically coherent high-risk subgroup. In the BCG-unresponsive setting, pembrolizumab (KEYNOTE-057) and nadofaragene firadenovec (QUILT-3.032) are guideline-endorsed treatment options [6,7]. Whether baseline morphological risk tier at the time of initial TURBT predicts differential response to these agents is unknown. The only way to answer that question properly is to pre-specify morphology-based risk stratification as a trial variable rather than add it retrospectively.

What is needed most immediately is a prospective multicentre study in BCG-treated T1HG NMIBC, powered for approximately 150 progression events, with substaging and budding thresholds defined before enrolment, formal inter-observer reliability assessment at site initiation, and re-TURBT substaging captured separately from the initial specimen. Until that study is performed, substaging and budding belong in pathology reports and MDT discussions as supporting information—not as independent triggers for RC, but also not as mere academic observations.

## Figures and Tables

**Table 1:** Summary of included studies evaluating tumour budding, T1 substaging, and CDH1 expression in T1HG NMIBC. Effect sizes are reported as provided in the original studies; NR indicates data not reported in accessible sources. Studies are ordered by marker domain. The evidence source for each study is indicated in the “Study design” column: entries marked “open full text” were extracted directly from the full publication, whereas the entry marked “abstract only” was extracted from the published abstract. “NR” (not reported) denotes a variable that was not available in the accessible source and reflects source completeness rather than a deficiency of the present analysis. Abbreviations: RFS, recurrence-free survival; HG-RFS, high-grade RFS; PFS, progression-free survival; CSS, cancer-specific survival; LP, lamina propria; MM, muscularis mucosae; BCG, bacillus Calmette-Guérin.

First Author & Year	Study Design	N	Population	Variable Assessed	Key Outcome	Main Result	Follow-Up	Major Limitation
Martin-Doyle 2015 [4]	Meta-analysis	15,215	High-grade T1	Selection factors for early RC incl. deep LP invasion	Outcomes in HG T1	Deep LP invasion had the largest adverse impact on outcomes; quantitative effect sizes not fully extractable from available abstract	NR	Heterogeneous retrospective source studies
Wan 2020 [5]	Systematic review	1516	High-grade NMIBC	Early vs. deferred cystectomy	CSS	Early cystectomy improved 5–10-yr CSS; RR 0.81 (*p* = 0.029)	NR	Retrospective source studies; heterogeneous definitions of early RC
Fukumoto 2016 [9]	Retrospective cohort	121	T1 bladder cancer	Tumour budding	Stage progress ion	5-yr PFS 91% budding-negative vs. 62% budding-positive; independent predictor on multivariable analysis	NR	Single-centre; bud-count threshold not validated externally
Eckstein 2021 [10]	Retrospective cohort (open full text)	92	pT1 NMIBC	Tumour budding (clusters ≤ 5 cells; median cut-off 0.9 buds per ×20 field )	RFS/PF S/CSS	Bud ≥ 0.9: worse RFS *p* = 0.005; PFS *p* = 0.017; CSS *p* = 0.002. BCG subgroup (*n* = 65): bud < 0.9 associated with zero progression events and no cancer-specific deaths	NR	Single-centre; retrospective; multivariable modelling event-limited
Busquets 2022 [14]	Retrospective cohort (abstract only)	95	pT1 high-grade	Tumour budding	Need for early RC	Budding associated with need for early RC; effect sizes not recoverable from abstract	NR	Abstract-only data; no full-text access
Parizi 2020 [11]	Systematic review + meta-analysis (open full text)	6781	T1/pT1 NMIBC	Substaging systems: MM-based and metric	Recurrence; progress ion	MM substaging: DR HR 1.23 (95%CI 1.01– 1.49), DP HR 2.61 (95%CI 1.61– 4.23). Depth/metric substaging subgroup: DR HR 1.49 (95%CI 1.11–2.00), DP HR 3.29 (95%CI 2.39–4.51). I^2^ = 47% for progression overall (random-effects); prediction intervals not reported	NR	Heterogeneous substaging definitions; no prediction intervals
de Jong 2021 [15]	Retrospective registry cohort	264	High-risk NMIBC ; ≥5 BCG instillations	Microinvasive vs. extensive LP invasion (centralised re-review)	BCG failure; HG-RFS; PFS	BCG failure 41% Vs. 21% (*p* = 0.002). 3-yr HG-RFS 64% vs 83% (*p* = 0.004). Multivariable: HG-RFS HR 3.2 (*p* = 0.005); PFS HR 3.0 (*p* = 0.009). Substaging discriminated PFS within highest EAU risk group (*p* = 0.038)	Median 68 mo	Retrospective; substaging inter-observer reliability not formally assessed
van de Putte 2018 [16]	Retrospective multicentre cohort	601	T1 bladder cancer	Metric substaging (0.5 mm threshold)	PFS; CSS	Metric substaging technically feasible in multicentre setting; independent prognostic value for PFS and CSS confirmed	NR	Effect sizes NR in accessible record
Soukup 2014 [17]	Retrospective cohort	200	T1 bladder cancer	pT1a vs. pT1b relative to MM	Prognosis	Depth of invasion relative to MM independently prognostic; effect sizes NR	NR	Requires MM identification; present in minority of specimens
Orsola 2015 [18]	Prospective single-centre cohort	132	High-grade T1	LP depth substaging strategy with two sequential BCG schedules	Recurrence; progress ion after BCG	LP depth substaging discriminated recurrence and progression risk regardless of MM visualisation status over 20-yr follow-up	20 yr	Single-centre; full-text quantitative data not accessible
Holmäng 1997 [19]	Prospective cohort	121	Primary T1	Depth of invasion	Prognosis	Invasion depth independently related to prognosis; quantitative details NR in accessible record	Long-term	Historical cohort; older management era

Abbreviations: BCG = bacillus Calmette–Guérin; CSS = cancer-specific survival; HG-RFS = high-grade recurrence-free survival; HR = hazard ratio; LP = lamina propria; MM = muscularis mucosae; NMIBC = non-muscle-invasive bladder cancer; NR = not reported; PFS = progression-free survival; RC = radical cystectomy; RFS = recurrence-free survival.

**Table 2:** Proposed framework for MDT discussion in T1HG NMIBC based on pathological risk features.

Clinical Scenario	Morphological Findings	MDT Discussion	Documentation
Extensive LP invasion AND high-grade budding	Both criteria met	Expedited MDT review recommended. Early RC should be discussed, formally documented, and offered within a shared decision-making framework [1,2,11,15].	Expedited
Extensive LP invasion alone	Substaging criterion met; budding low or absent	BCG induction and maintenance per EAU guidelines appropriate; note that extensive LP invasion is associated with a BCG failure rate approximately twice that of microinvasive disease [15], which should inform how closely this patient is monitored. Early repeat TURBT at 4–6 wk mandatory. Revisit escalation at first high-grade recurrence [1,2,15].	Standard MDT
Microinvasive T1, low budding	No criteria met	Guideline-concordant BCG pathway is supported by available morphological data. Standard EAU surveillance schedule applies [1,2].	Routine

Abbreviations: BCG = bacillus Calmette-Guérin; EAU = European Association of Urology; HG = high-grade; LP = lamina propria; MDT = multidisciplinary team; RC = radical cystectomy; TURBT = transurethral resection of bladder tumour.

## Data Availability

No new data were created or analysed in this study. All data supporting the findings of this review are derived from published sources cited in the reference list.

## Supplementary Materials

The following supporting information can be downloaded at: https://www.mdpi.com/article/10.3390/cancers18142269/s1. File S1: Full PubMed/MEDLINE search string; File S2: QUIPS/AMSTAR-2 quality appraisal table; File S3: SANRA quality assessment and PRISMA-adapted study selection flow.
