# Supplementary material for: Low-Cost Pathology Signals for Risk Stratification in High-Risk Non-Muscle-Invasive Bladder Cancer: A Narrative Review
Source: Cancers (Basel). 2026 Jul 15;18(14):2269. doi: 10.3390/cancers18142269 (PMC13407352; doi:10.3390/cancers18142269)
Supplement: Supplementary file 1 [file cancers-18-02269-s001.zip › File S1. SearchString.pdf]

Low-Cost Pathology Signals for Risk Stratification in High-Risk NMIBC

Supplementary File S1 — Full PubMed/MEDLINE Search String

Database: PubMed/MEDLINE

Date of search: 27 February 2026

Date range: 1 January 2000 – 27 February 2026

Language restriction: English

Human studies only: Yes (filter applied post-retrieval)

Excluded publication types: Letters, editorials, comments (filter applied post-retrieval)

Full Boolean Search String

Block 1 — Population:

("urinary bladder neoplasms"[MeSH] OR "non-muscle-invasive bladder cancer"[tiab] OR "NMIBC"[tiab] OR "T1 urothelial carcinoma"[tiab] OR "T1 bladder cancer"[tiab] OR "T1 bladder carcinoma"[tiab] OR "high-grade T1 bladder"[tiab] OR "pT1 urothelial"[tiab])

AND

Block 2 — Biomarker domains:

("tumor budding"[tiab] OR "tumour budding"[tiab] OR "tumor bud"[tiab] OR "tumour bud"[tiab] OR "T1 substaging"[tiab] OR "substaging"[tiab] OR "lamina propria invasion"[tiab] OR "muscularis mucosae"[tiab] OR ("lamina propria"[tiab] AND "depth"[tiab]) OR "microinvasive"[tiab] OR "extensive invasion"[tiab] OR "metric substaging"[tiab] OR "E-cadherin"[tiab] OR "CDH1"[tiab] OR "CDH1 protein"[nm] OR "N-cadherin"[tiab] OR "CDH2"[tiab] OR "P-cadherin"[tiab] OR "CDH3"[tiab] OR "cadherin switching"[tiab] OR "cadherin switch"[tiab] OR ("catenin"[tiab] AND ("bladder"[tiab] OR "urothelial"[tiab])) OR "epithelial-mesenchymal transition"[MeSH] OR "pEMT"[tiab])

AND

Block 3 — Oncological endpoints:

("recurrence"[tiab] OR "progression"[tiab] OR "BCG failure"[tiab] OR "BCG-unresponsive"[tiab] OR "BCG-refractory"[tiab] OR "BCG unresponsive"[tiab] OR "radical cystectomy"[tiab] OR "cancer-specific survival"[tiab] OR "progression-free survival"[tiab] OR "disease-specific survival"[tiab] OR "recurrence-free survival"[tiab] OR "RFS"[tiab])

Study selection figures

Records identified in database search: 214

Records identified via manual reference tracking: 3

Total records identified: 217

Duplicates removed: 0

Records excluded at title/abstract screening: 141

Full texts assessed for eligibility: 76 (73 from database + 3 from manual tracking)

Full texts excluded with reasons: 48 (see Appendix C)

Studies included in narrative synthesis: 28
