# Supplementary material for: Low-Cost Pathology Signals for Risk Stratification in High-Risk Non-Muscle-Invasive Bladder Cancer: A Narrative Review
Source: Cancers (Basel). 2026 Jul 15;18(14):2269. doi: 10.3390/cancers18142269 (PMC13407352; doi:10.3390/cancers18142269)
Supplement: Supplementary file 1 [file cancers-18-02269-s001.zip › File S2. QUIPS AMSTAR2 2.pdf]

Low-Cost Pathology Signals for Risk Stratification in High-Risk NMIBC

Supplementary File S2 — QUIPS/AMSTAR-2 quality appraisal table

Section 1. Risk of Bias in Prognostic Cohort Studies — QUIPS

QUIPS tool (Hayden et al., Ann Intern Med. 2013;158:280–286). Six domains rated: L = Low; M = Moderate; H = High risk of bias; NI = No information (abstract-only record). Colour coding applied per domain rating.

Study	D1 Participation	D2 Attrition	D3 Factor meas.	D4 Outcome meas.	D5 Confounding	D6 Analysis	Overall Risk of Bias
**Fukumoto 2016 [9]**	M	M	M	L	M	M	**Moderate**
**Eckstein 2021 [10]**	M	L	M	L	M	L	**Moderate**
**Busquets 2022 [15]**	NI	NI	NI	NI	NI	NI	**NI — abstract only**
**de Jong 2021 [16]**	L	L	L	L	L	L	**Low**
**van de Putte 2018 [17]**	M	L	L	L	M	M	**Moderate**
**Soukup 2014 [18]**	M	M	M	L	M	M	**Moderate**
**Orsola 2015 [19]**	L	L	L	L	L	M	**Low–Moderate**
**Holmäng 1997 [20]**	**H**	M	M	L	**H**	M	**High**

Domain definitions:

D1 Participation: consecutive sampling and explicit inclusion criteria. D2 Attrition: completeness of follow-up. D3 Factor measurement: bud-count or substaging threshold derivation in same cohort = M. D4 Outcome measurement: endpoint definition and blinding. D5 Confounding: multivariable adjustment for CIS, multifocality, prior BCG. D6 Statistical analysis: appropriate model selection, event count.

Reference: Hayden JA, van der Windt DA, Cartwright JL, Côté P, Bombardier C. Assessing bias in studies of prognostic factors. Ann Intern Med. 2013;158(4):280–286. doi:10.7326/0003-4819-158-4-201302190-00009

Section 2. Methodological Quality of Included Meta-Analyses — AMSTAR-2

AMSTAR-2 (Shea et al., BMJ 2017;358:j4008). Critical domains (†) shown only. Y = Yes; PY = Partial Yes; N = No. Per AMSTAR-2: ≥1 critical domain with N or PY = Critically Low overall confidence.

Study	Item 2† (Protocol)	Item 4† (Search)	Item 7† (Excl. list)	Item 9† (RoB assess.)	Item 11† (Methods)	Item 15† (Pub. bias)	Overall Confidence
**Martin-Doyle 2015 [4]**	**N**	PY	**N**	**N**	PY	**N**	**Critically Low**
**Wan 2020 [5]**	**N**	PY	**N**	**N**	PY	**N**	**Critically Low**
**Parizi 2020 [11]**	**N**	PY	PY	PY	PY	PY	**Critically Low***
**Xie 2016 [28]**	**N**	PY	PY	**N**	PY	**N**	**Critically Low****

Footnotes:

* Parizi 2020 [11]: Item 2† N (no prospective protocol registration); items 4†, 7†, 9†, 11†, 15† all PY. Per AMSTAR-2, more than one critical domain weakness = Critically Low. The pooled HR of 3.29 should be read as a directional signal, not a calibrated, cross-centre threshold. This limitation is stated explicitly in the Limitations section of the main manuscript.

** Xie 2016 [28]: Items 9† (risk of bias in included studies not assessed) and 15† (publication bias not investigated) both N = two critical domain failures = Critically Low. Pooled ORs used as hypothesis-generating directional evidence only.

Martin-Doyle 2015 [4] and Wan 2020 [5]: accessed at abstract level only; multiple AMSTAR-2 domains could not be assessed. Both are used for clinical context (RC timing outcomes), not as primary evidence for the biomarker parameters under review.

† Critical AMSTAR-2 domains: 2 (protocol registration); 4 (comprehensive search, ≥2 databases); 7 (list of excluded studies with justification); 9 (risk of bias in included studies assessed); 11 (appropriate meta-analytic methods); 15 (publication bias investigated).

Reference: Shea BJ, Reeves BC, Wells G, et al. AMSTAR 2: a critical appraisal tool for systematic reviews. BMJ. 2017;358:j4008. doi:10.1136/bmj.j4008
