# Supplementary material for: Low-Cost Pathology Signals for Risk Stratification in High-Risk Non-Muscle-Invasive Bladder Cancer: A Narrative Review
Source: Cancers (Basel). 2026 Jul 15;18(14):2269. doi: 10.3390/cancers18142269 (PMC13407352; doi:10.3390/cancers18142269)
Supplement: Supplementary file 1 [file cancers-18-02269-s001.zip › File S3. SANRA PRISMA.pdf]

Low-Cost Pathology Signals for Risk Stratification in High-Risk NMIBC

Supplementary File S3 — SANRA Quality Assessment and PRISMA-Adapted Study Selection Flow

Part 1. SANRA Quality Assessment

SANRA (Baethge et al., Res Integr Peer Rev 2019) is the quality framework for this narrative review. Six criteria scored 0–2; maximum score 12.

1. Justification of importance: 2/2 — *Introduction establishes 10–40% progression rate and unmet stratification need.*

2. Aims / questions: 2/2 — *Three pre-specified clinical questions stated in Methods.*

3. Literature search: 1/2 — *Single database (PubMed/MEDLINE). Limitation stated in Methods and Limitations.*

4. Referencing: 2/2 — *All quantitative claims carry numbered citations with effect estimates.*

5. Scientific reasoning: 2/2 — *CDH1/pEMT mechanistic framework developed; prognostic vs predictive distinction explicit.*

6. Balanced view: 2/2 — *I^2 values reported; budding reproducibility gap identified; CDH1 IHC non-actionable conclusion.*

Total SANRA score: 11/12. Criterion 3 rated 1/2 (single-database search). All other criteria at maximum.

Reference: Baethge C, Goldbeck-Wood S, Mertens S. SANRA. Res Integr Peer Rev. 2019;4:5. doi:10.1186/s41073-019-0064-8

Part 2. Study Selection Flow (PRISMA-adapted for narrative synthesis)

Identification: *PubMed/MEDLINE (n=214) + manual tracking (n=3) = 217 total. Duplicates: 0.*

Screening: *217 screened. Excluded 141 (irrelevant topic 68; wrong stage/type 35; non-English 12; case series 15; duplicate abstracts 11). Proceeding: 76.*

Eligibility: *76 full texts assessed. Excluded 48 (no T1HG outcome 18; no effect estimate 14; n<30 8; review/editorial 5; inaccessible full text 3). Included: 28.*

Included: *28 studies. Tumour budding n=3; T1 substaging n=7; CDH1/adhesion biology n=10; EAU guidelines n=3; NMIBC clinical context n=5. Open full text n=19; abstract only n=9.*

Note: All stages performed by single reviewer. See Figure 1 in main manuscript for flow diagram.

Adapted from: Page MJ et al. PRISMA 2020. BMJ. 2021;372:n71. doi:10.1136/bmj.n71
